# Severe hyperparathyroidism is associated with nutritional impairment in maintenance hemodialysis patients

**DOI:** 10.3389/fnut.2022.933918

**Published:** 2022-09-13

**Authors:** Sinee Disthabanchong, Kornpong Vantanasiri, Sirote Khunapornphairote, Payupol Chansomboon, Nuchcha Buachum, Sarunya Saeseow

**Affiliations:** ^1^Division of Nephrology, Department of Medicine, Faculty of Medicine, Ramathibodi Hospital, Mahidol University, Bangkok, Thailand; ^2^Faculty of Medicine, Siriraj Hospital, Mahidol University, Bangkok, Thailand; ^3^Faculty of Medicine, Khon Kaen University, Khon Kaen, Thailand

**Keywords:** ESRD, ESKD, dialysis, sarcopenia, nutrition, hypoalbuminemia

## Abstract

Severe hyperparathyroidism predicts poor outcomes in patients with kidney failure. Mechanisms underlying the relationship between high parathyroid hormone (PTH) and decreased survival other than bone loss are largely unexplored. Recent evidence suggests the role of excess PTH in adipose tissue browning resulting in protein-energy wasting. The present retrospective observational study examined nutritional status among patients receiving maintenance hemodialysis with different degree of hyperparathyroidism. Seven hundred forty-five patients were categorized into four groups according to PTH levels: group 0, < 200; group 1, 200–599; group 2, 600–1,499; and group 3, ≥1,500 pg/ml. Group 0 was excluded because of the relationship between low PTH with aging and malnutrition. Patients in groups 1 and 2 were matched to group 3 by propensity score yielding 410 patients in the final analysis. Nutritional parameters at baseline and the preceding 1 and 2 years were examined. At baseline, lower serum albumin, creatinine/body surface area (Cr/BSA), height in female and higher percentage of patients with serum albumin < 38 g/L were observed in group 3 compared to groups 1 and 2. Higher PTH level was independently associated with serum albumin < 38 g/L and Cr/BSA < 380 μmol/L/m^2^. The longitudinal decline in serum albumin and Cr/BSA and the increase in the frequency of patients with serum albumin < 38 g/L were observed among patients in group 3. Between group comparisons confirmed a significant decline in serum albumin and Cr/BSA in association with an increase in the proportion of patients with serum albumin < 38 g/L and Cr/BSA < 380 μmol/L/m^2^ in group 3 compared to groups 1 and 2. Weight loss was more significant and was of greater magnitude among patients in group 3 compared to groups 1 and 2. Normalized protein catabolic rate in 3 groups were comparable. There was no significant difference in any of the nutritional parameters between groups 1 and 2. In conclusion, patients receiving maintenance hemodialysis with severe hyperparathyroidism showed deterioration of nutritional status compared to patients with moderate hyperparathyroidism and patients with PTH level in the recommended range. These findings support the role of extreme PTH level in protein-energy wasting emphasizing the importance of early management of hyperparathyroidism.

## Introduction

An increase in parathyroid hormone (PTH) level occurs as early as chronic kidney disease (CKD) stage 3 as a result of a decrease in the availability of 1,25-dihydroxyvitamin D. In later stages, phosphate retention stimulates the production and the release of PTH from parathyroid glands by yet unknown mechanisms. As CKD advances, the severity of hyperparathyroidism amplifies, and parathyroid glands become enlarged (1). At this stage, it becomes increasingly difficult to control PTH level and some patients start experiencing physical disability from high turnover bone disease and muscle weakness. Extreme PTH levels have consistently been shown to predict increased mortality risk among patients with kidney failure (2–5). Mechanisms underlying the relationship between high PTH level and poor outcomes other than bone loss have not been clearly elucidated. A decrease in serum albumin associated with severe hyperparathyroidism and its improvement after parathyroidectomy has been reported in previous small studies (6–8). Others also suggested an increase in resting energy expenditure and muscle wasting in severe hyperparathyroidism (9, 10). A recent large epidemiological study demonstrated a relationship between increased PTH level and 12-month weight loss in patients receiving maintenance hemodialysis (MHD) (11). These data suggested that severe hyperparathyroidism in dialysis patients could result in nutritional impairment. The present study examined nutritional status in patients receiving MHD with different degree of hyperparathyroidism.

## Materials and methods

### Study design and setting

This retrospective study was approved by Human Research Ethics Committee of Faculty of Medicine, Ramathibodi Hospital, Mahidol University, Bangkok, Thailand (approval number MURA2021/774 and MURA2017/220) and was conducted according to the Declaration of Helsinki. The study has been granted an exemption from requiring written informed consent.

### Participants

**FIGURE 1:**
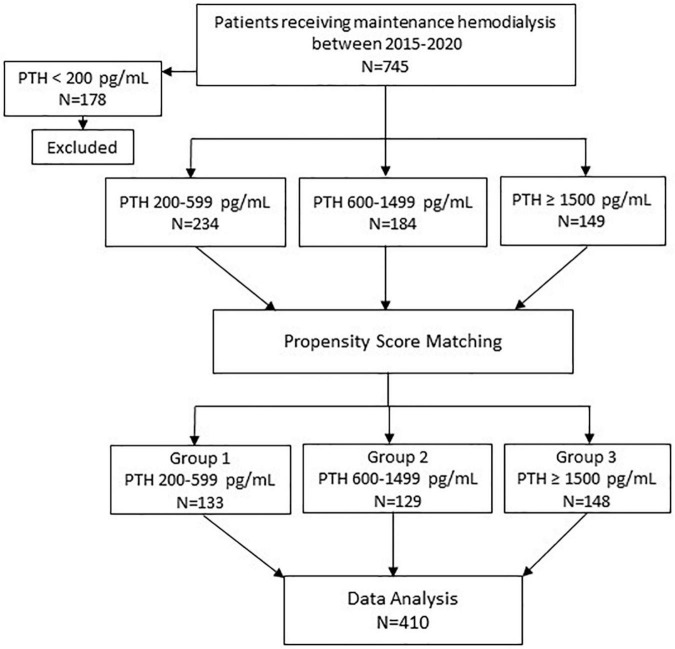
Study flow chart. PTH, parathyroid hormone.

The details on patient selection are shown in Figure 1. All patients receiving MHD between 2015 and 2020 at Ramathibodi hospital were identified. The inclusion criteria were: (1) age ≥ 18 years; (2) having been on MHD for ≥ 12 months. Patients who had parathyroidectomy were excluded. Seven hundred forty-five patients met the eligibility criteria. To categorize the patients according to the degree of hyperparathyroidism, PTH values were stratified into the following ranges; < 200 pg/ml (group 0); 200–599 pg/ml (group 1); 600–1,500 pg/ml (group 2); and ≥ 1,500 pg/ml (group 3). The recommended PTH level according to the guidelines published by Kidney Disease: Improving Global Outcomes (KDIGO) for patients receiving MHD was 2–9 times of the upper limit of normal which corresponded to the PTH range of group 1 (12). Groups 2 and 3 represented moderate and severe hyperparathyroidism, respectively. PTH values during the previous 12 months prior to enrollment for each patient were reviewed and the patient was categorized to the group into which most of their PTH values fell. Among those who died, PTH values during 12–24 months prior to death were used. Patients in Group 0 were excluded because they were older and more likely to have chronic illnesses and malnutrition (13). Patients in group 3 were younger, less likely to have diabetes and had longer dialysis vintage compared to patients in groups 1 and 2. To reduce potential biases from unbalanced baseline characteristics, separate pairwise propensity score matchings of patients in group 1 to group 3 and group 2 to group 3 were performed. Group 3 was chosen as a reference group for matching because group 3 had the lowest number of patients. The matching yielded 133 patients from group 1 and 129 patients from group 2. The 148 patients from group 3 who were matched to patients from either group 1 or 2 were included in analyses. All patients received 4-h hemodialysis session using high flux dialyzer, 2–3 times/week, with dialysate calcium concentration ranging between 2.5 and 3.5 mEq/L.

### Outcomes and predictors

Outcomes were as follows: the difference in parameters of nutritional status including body weight, body mass index (BMI), normalized protein catabolic rate (nPCR), serum albumin and creatinine/body surface area (Cr/BSA) at baseline between three groups of patients, the difference in the longitudinal changes of these nutritional parameters over the 36-month observational period, and the factors predicting nutritional impairment.

### Nutritional assessment

To evaluate nutritional status, the criteria proposed by the International Society of Renal Nutrition and Metabolism (ISRNM) for protein-energy wasting (PEW) including serum albumin as serum biochemistry, BMI as body mass, serum Cr/BSA as skeletal muscle mass, and nPCR as protein intake was adapted. The cut-off values are as follow: nPCR 0.8 g/kg/day, serum albumin 38 g/L, serum Cr/BSA 380 μmol/L/m^2^ (14, 15). Due to small body size of the study population, the cut-off value of < 18 kg/m^2^ for the BMI was selected based on its association with increased mortality in Japanese hemodialysis patients (16, 17). BSA was calculated using DuBois & DuBois equation: 0.20247 × height (m)^0^.^725^ × weight (kg)^0^.^425^. Body weight and height were obtained from the records of outpatient clinic visits on non-dialysis days. The data on nPCR and Kt/V were obtained from Thailand Renal Replacement Therapy Registry. Electronic records of hemodialysis treatment were available from 2018 onward. The data on Kt/V and nPCR were available in 204 patients for baseline comparisons. The data for the preceding 12–24 months were available in 139 patients. Longitudinal data up to 36 months prior to enrollment were only available in 61 patients and was excluded from analyses. Serum cholesterol was excluded due to the interference from lipid-lowering agents.

### Biochemical data

Demographics and laboratory data were obtained from electronic medical records. Neutrophil-to-lymphocyte ratio (NLR) and platelet-to-lymphocyte ratio (PLR) were selected as markers of inflammation because the data were available in all patients (18, 19). NLR ≥ 3.5 and PLR ≥ 140 have been shown to indicate significant inflammation and predict mortality in patients receiving MHD (20–22). Serum calcium was corrected based on the following equation: corrected calcium (mg/dl) = serum calcium (mg/dl) + [(40 - serum albumin (g/l))/10 × 0.8]. PTH was measured by intact PTH assay (Elecsys STAT intact PTH assay).

The six-month average values of body weight, height and laboratory data were used to analyze the differences in biochemical and nutritional data at baseline. To investigate the longitudinal changes, 12-month average values were used. The duration of the study was divided into three periods including Year 0 (0 to –12 months), Year –1 (–12 to –24 months) and Year –2 (–24 to –36 months). For every 12-month period, the average body weight, height, and laboratory data were calculated. Body weight and laboratory data obtained during acute illnesses were excluded.

### Statistical analysis

Data are presented as mean ± standard deviation or median (interquartile range). Propensity score matching was performed according to age, sex, diabetes and dialysis vintage with a matching tolerance of 0.05 and maximized performance and without replacement. Differences in baseline biochemical and nutritional parameters were analyzed by one-way ANOVA, Chi-square, Bonferroni or Kruskal–Wallis one-way ANOVA tests where appropriate. To determine factors associated with nutritional impairment, univariate and multivariate generalized linear regression models were applied. The multivariate model consisted of variables with *p*-value < 0.1 from the univariate model. Mixed-effects regression analysis was used to determine the longitudinal changes of nutritional parameters. The correlation between two continuous variables was performed with Pearson’s correlation. *P*-value < 0.05 was considered statistically significant. All statistical analyses were performed using IBM SPSS Statistics for Windows, version 26 (IBM Corp., Armonk, NY, United States).

## Results

Study flow chart is shown in Figure 1. A total of 410 patients including 133 patients in group 1 (PTH 200–599 pg/ml), 129 patients in group 2 (PTH 600–1,499 pg/ml) and 148 patients in group 3 (PTH ≥ 1,500 pg/ml) were available for analysis.

### Baseline biochemical and nutritional data

**FIGURE 2:**
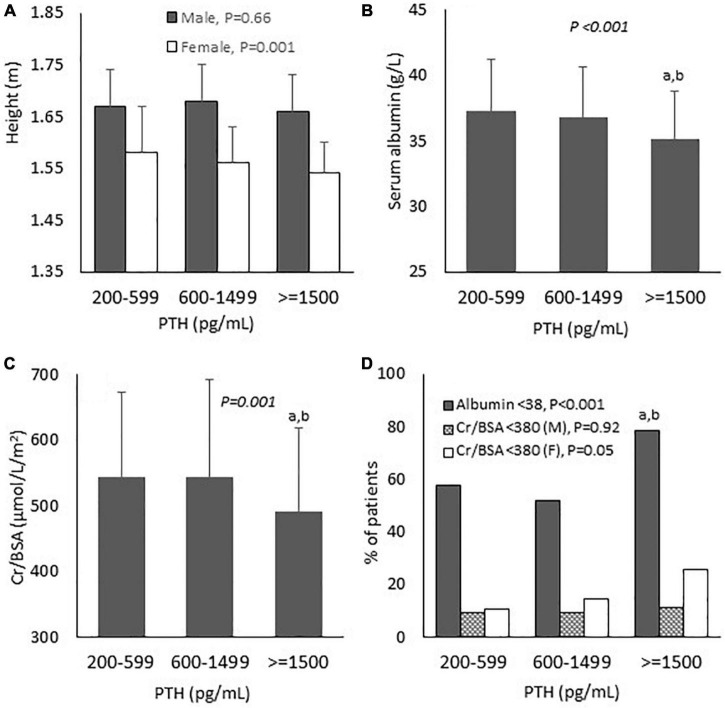
Baseline biochemical and nutritional parameters according to parathyroid hormone levels. **(A)** Height; **(B)** Serum albumin; **(C)** Serum creatinine/body surface area; and **(D)** Proportion of patients with nutritional impairment. PTH, parathyroid hormone; Cr, creatinine; BSA, body surface area; M, male; F, female. *P*-values in the graphs represent the significant difference between all groups. *^a^P* < 0.01 vs. PTH 200–599; *^b^P* < 0.01 vs. PTH 600–1,499.

Comparisons of baseline biochemical and nutritional data are shown in Table 1 and Figure 2. All groups were largely equivalent in age, sex, body weight, underlying diseases, dialysis vintage, the frequency and the adequacy of hemodialysis. With increasing severity of hyperparathyroidism, hemoglobin became lower, whereas serum calcium, phosphate and proportions of patients receiving active vitamin D and calcimimetics for hyperparathyroidism rose significantly. The inflammatory markers including NLR and PLR in all groups were comparable. Lower height and BSA were observed only among females. Pairwise comparisons revealed lower height and BSA among females in group 3 compared to group 1. Lower serum albumin, creatinine and Cr/BSA and higher percentage of patients with serum albumin < 38 g/L were noted. Pairwise comparisons revealed substantially lower serum albumin, creatinine and Cr/BSA and higher percentage of patients with serum albumin < 38 g/L in group 3 compared to groups 1 and 2. More female patients seemed to have serum Cr/BSA < 380 μmol/L/m^2^ but the difference did not reach statistical significance. Body weight, BMI and nPCR in all groups were comparable. Pairwise comparisons did not show any significant differences in nutritional data between patients in groups 1 and 2

**TABLE 1:** Baseline biochemical and nutritional parameters according to parathyroid hormone levels.

Parameters	Parathyroid hormone (pg/ml)			*P*
	Group 1 PTH 200–599 (*n* = 133)	Group 2 PTH 600–1,499 (*n* = 129)	Group 3 PTH ≥ 1,500 (*N* = 148)	
**Baseline parameters**				
Age (year)	49.26 ± 14.4	46.28 ± 13.28	46.04 ± 12.22	0.09
Male (*n*/%)	65 (48.9)	67 (51.9)	77 (52)	0.84
Body weight (kg)	62.03 ± 13.43	62.9 ± 18	61.11 ± 14.8	0.63
Male	65.92 ± 12.32	70.02 ± 19.78	67.35 ± 14.45	0.32
Female	58.31 ± 13.48	55.2 ± 11.87	54.43 ± 12.05	0.16
Height (cm)	162.3 ± 8.93	161.9 ± 8.84	160 ± 8.88	0.06
Male	166.5 ± 7.24	167 ± 6.95	165.9 ± 6.68	0.66
Female	158.4 ± 8.6	156.4 ± 7.3	153.6 ± 6.13*^b^*	0.001
Body surface area (m^2^)	1.66 ± 0.19	1.66 ± 0.23	1.62 ± 0.25	0.2
Male	1.73 ± 0.17	1.77 ± 0.23	1.72 ± 0.27	0.33
Female	1.58 ± 0.19	1.53 ± 0.17	1.5 ± 0.16*^a^*	0.03
Diabetes (*n*/%)	21 (15.8)	14 (10.9)	15 (10.1)	0.3
Cardiovascular disease (*n*/%)	21 (15.8)	17 (13.2)	24 (16.2)	0.75
Hypertension (*n*/%)	118 (88.7)	113 (87.6)	128 (86.5)	0.85
Phosphate binders (yes/no)	111 (84.7)	91 (71.1)*^a^*	110 (74.3)	0.02
Active vitamin D (yes/no)	66 (49.6)	71 (55)	99 (66.9)*^a^*	0.01
Calcimimetics (yes/no)	17 (12.9)	17 (13.2)	36 (24)*^a^*	0.02
Dialysis vintage (months)	72.8 ± 55.1	70.2 ± 41.9	77.9 ± 41.7	0.37
Twice weekly HD (*n*/%)	33 (24.8)	27 (20.9)	22 (15.1)	0.12
Kt/V	1.86 ± 0.34	1.78 ± 0.37	1.88 ± 0.4	0.32
Hemoglobin (g/dl)	10.8 ± 1.66	11.15 ± 1.58	10.42 ± 1.57*^e^*	0.001
Neutrophil-lymphocyte ratio	3.5 ± 1.7	3.26 ± 1.55	3.52 ± 1.93	0.43
Platelet-lymphocyte ratio	183 ± 80	170 ± 70	185 ± 79	0.29
Calcium (mg/dl)	9.88 ± 0.89	10.08 ± 0.91	10.5 ± 0.82^c,f^	<0.001
Phosphate (mg/dl)	5.15 ± 1.34	5.48 ± 1.4	5.72 ± 1.28*^b^*	0.002
Parathyroid hormone (pg/ml)	371 (307–457)	976 (731–1,156)*^c^*	2,272 (1,841–2,957)*^c^*,*^f^*	<0.001
Creatinine (mg/dl)	10.13 ± 2.54	10.2 ± 3	9.01 ± 2.6*^b^*,*^e^*	<0.001
**Nutritional parameters**				
Body mass index (kg/m^2^)	23.45 ± 4.29	23.78 ± 5.42	23.73 ± 4.7	0.83
nPCR (g/kg/day)	1.26 ± 0.26	1.26 ± 0.29	1.24 ± 0.27	0.9
Albumin (g/L)	37.11 ± 3.78	37.31 ± 3.93	35.5 ± 3.62*^b^*,*^f^*	<0.001
Cr/BSA (μmol/L/m^2^)	543.6 ± 128.9	543.7 ± 148.5	491.3 ± 127.4*^b^*,*^e^*	0.001
**ISRNM criteria for PEW (*n*/%)**				
Body mass index < 18 kg/m^2^	7 (5.3)	9 (7)	11 (7.5)	0.74
nPCR < 0.8 g/kg/day	2 (2.1)	3 (3.9)	1 (2.3)	0.75
Albumin < 38 g/L	76 (57.6)	67 (51.9)	116 (78.4)*^c^*,*^f^*	<0.001
Cr/BSA < 380 μmol/L/m^2^	13 (9.8)	15 (11.6)	26 (17.9)	0.11
Male	6 (9.2)	6 (9)	8 (10.8)	0.92
Female	7 (10.4)	9 (14.5)	18 (25.4)	0.05

Data are present as mean ± standard deviation or median (interquartile range); PTH, parathyroid hormone; HD, hemodialysis; nPCR, normalized protein catabolic rate; Cr, creatinine; BSA, body surface area; ISRMN, International Society of Renal Nutrition and Metabolism; PEW, protein energy wasting.

*^a^p* < 0.05; *^b^p* < 0.01; *^c^P* < 0.001 vs. PTH 200–600; *^d^p* < 0.05; *^e^p* < 0.01, *^f^p* < 0.001 vs. PTH 600–1,500.

### Factors associated with nutritional impairment

Pearson’s correlations of biochemical and nutritional parameters are shown in Supplementary Table 1. BMI was positively associated with hemoglobin and serum creatinine and negatively associated with PTH. There were no correlations between nPCR with any of the biochemical or nutritional parameters. Serum albumin showed positive correlations with hemoglobin and serum creatinine and negative correlations with age, NLR, PLR and PTH. Serum creatinine showed positive correlations with BMI, serum albumin and serum phosphate and negative correlations with age, PLR and PTH. Analyses of factors associated with nutritional impairment by generalized linear model are shown in Table 2. Increasing age, the presence of cardiovascular disease and NLR ≥ 3.5 and higher PTH level were independently associated with serum albumin < 38 g/L. Increasing age, lower serum phosphate and higher PTH level were independently associated with serum Cr/BSA < 380 μmol/L/m^2^.

**TABLE 2:** Generalized linear models of factors associated with nutritional impairment at baseline.

Factors	Serum albumin < 38 g/L				Serum Cr/BSA < 380 μmol/L/m^2^			
	Univariate		Multivariate		Univariate		Multivariate	
	β	*P*	β	*P*	β	*P*	β	*P*
	(95% CI)		(95% CI)		(95% CI)		(95% CI)	
Age (1 year)	0.009(0.006,0.013)	<0.001	0.008(0.004, 0.01)	<0.001	0.004(0.002, 0.007)	0.001	0.003(0.000,0.005)	0.04
Male sex	–0.008(–0.18, 0.02)	0.18	–	–	-0.07(–0.13, –0.002)	0.04	–0.05(-0.11, 0.01)	0.13
Diabetes	0.1(-0.04, 0.26)	0.17	–	–	0.04(-0.06, 0.14)	0.4	–	–
CVD	0.32(0.18, 0.45)	<0.001	0.19(0.06, 0.32)	0.003	0.08(-0.01, 0.17)	0.09	0.04(-0.06, 0.13)	0.44
Thrice weekly HD	0.2(0.06, 0.32)	0.001	0.1(-0.01, 0.21)	0.09	0.003(-0.08, 0.09)	0.94	–	–
DV (1 year)	0.02(0.007, 0.03)	0.002	0.01(-0.001, 0.02)	0.09	0.005(-0.003, 0.01)	0.2	–	–
NLR ≥ 3.5	0.2(0.1, 0.3)	<0.001	0.18(0.09, 0.27)	<0.001	0.05(-0.02, 0.12)	0.18	–	–
PLR ≥ 140	0.008(-0.1, 0.11)	0.89	–	–	–0.02(-0.09, 0.05)	0.57	–	–
Phosphate (1 mg/dl)	0.01(-0.02, 0.05)	0.48	–	–	–0.05(-0.07, –0.02)	<0.001	–0.04(-0.07, –0.02)	0.001
PTH (100 pg/ml)	0.01(0.008, 0.02)	<0.001	0.01(0.008, 0.02)	<0.001	0.004(0.001, 0.008)	0.01	0.006(0.002, 0.009)	0.001

ß, regression coefficient; CI, confidence interval; Cr, creatinine; BSA, body surface area; CVD, cardiovascular disease; HD, hemodialysis; DV, dialysis vintage; NLR, neutrophil-to-lymphocyte ratio; PLR, platelet-to-lymphocyte ratio; PTH, parathyroid hormone.

### Longitudinal changes of nutritional parameters

**FIGURE 3:**
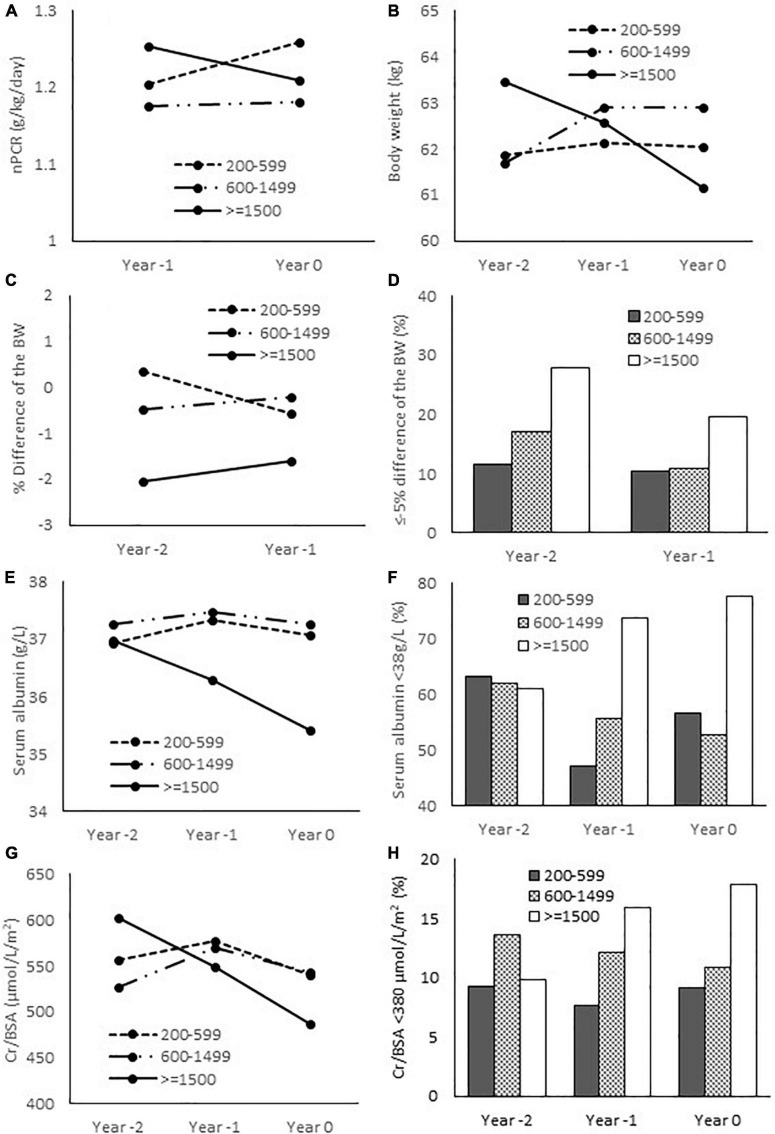
Nutritional parameters at baseline and the preceding 1 and 2 years. **(A)** Normalized protein catabolic rate; **(B)** Body weight; **(C)** Relative difference of the body weight from baseline (Year 0); **(D)** Proportion of patients with ≤ -5% difference of the body weight from baseline; **(E)** Serum albumin; **(F)** Proportion of patients with serum albumin < 38 g/L; **(G)** Serum creatinine/body surface area; **(H)** proportion of patients with serum Cr/BSA < 380 μmol/L/m^2^. nPCR, normalized protein catabolic rate; BW, body weight; Cr, creatinine; BSA, body surface area.

To further explore the longitudinal changes of nutritional parameters in relation to the degree of hyperparathyroidism, the data on body weight, height, nPCR, serum albumin and creatinine during the preceding 2 years were collected. Table 3, Figure 3 and Supplementary Table 2 showed the results of mixed-effects regression analysis. There was no significant difference in the body weight and the BMI between baseline (Year 0) compared to the preceding 1 (Year –1) and 2 years (Year –2) in any group (Supplementary Table 2). Between group comparisons also yielded negative results. Height loss was significant only among females in group 3 compared to group 1. Changes in the body weight were further analyzed as the percentage change from baseline (Table 3 and Figure 3). Weight reduction from the previous 2 years was more substantial in group 3 compared to group 2. More patients in group 3 had ≥ 5% weight loss compared to groups 1 and 2. The changes in nPCR from the previous year in all groups were negligible. There was no significant difference in the change of nPCR between the 3 groups. A substantial decrease in serum albumin with an increase in the proportion of patients with serum albumin < 38 g/L from the previous 2 years was observed among patients in group 3. Between group analyses confirmed a significant decline in serum albumin and an increase in the proportion of patients with serum albumin < 38 g/L from the preceding 1 and 2 years among patients in group 3 compared to groups 1 and 2. Serum Cr/BSA decreased substantially from the previous 2 years among patients in group 3. Between group analyses confirmed a significant decline in serum Cr/BSA from the preceding 1 and 2 years in group 3 compared to groups 1 and 2. The proportion of patients with serum Cr/BSA < 380 μmol/L/m^2^ increased substantially from the preceding 1 and 2 years among patients in group 3 compared to group 1. Supplementary Table 2 showed serum Cr/BSA in males and females. An increase in the proportion of patients with serum Cr/BSA < 380 μmol/L/m^2^ from the preceding 1 and 2 years was observed only among females in group 3 compared to groups 1 and 2. There was no difference in any of the nutritional parameters between patients in groups 1 and 2.

**TABLE 3:** Mixed-effects regression analyses on the differences in nutritional parameters between baseline and the preceding 1 and 2 years.

Parameters	Time	Mean (95% confidence Interval)			Within group difference from Year 0 Mean (95% confidence interval)			Between group difference Mean (95% confidence interval)					
								Comparator group 1				Comparator group 2	
		Group 1	Group 2	Group 3	Group 1	Group 2	Group 3	Group 2	*P*	Group 3	*P*	Group 3	*P*
		200–599	600–1,499	≥1,500	200–599	600–1,499	≥1,500	600–1,499		≥1,500		≥1,500	
Difference of the BW from Year 0 (%)	Year -1	–0.56 (-1.3, 0.18)	–0.21 (-1.06, 0.65)	–1.61 (-2.88, –0.34)	–	–	–	–	–	–	–	–	–
	Year -2	0.36 (-0.42, 1.14)	–0.48 (-1.41, 0.46)	–2.06 (-3.48, –0.64)	–0.92 (-2, 0.15)	0.27 (-1, 1.54)	0.45 (-1.46, 2.35)	–0.36 (-1.5, 0.77)	0.53	0.95 (-0.19, 2.09)	0.1	1.31 (0.16, 2.46)	0.03
≤-5% difference of the BW from Year 0 (*n*/%)	Year –1	13 (10.5)	13 (10.9)	22 (19.5)	–	–	–	–	–	–	–	–	–
	Year –2	13 (11.6)	17 (17.2)	25 (27.8)	–0.01 (-0.09, 0.07)	–0.06 (-0.16, 0.03)	–0.08 (-0.2, 0.03)	–0.02 (-0.1, 0.06)	0.57	–0.11 (-0.19, –0.03)	0.005	–0.09 (-0.17, –0.01)	0.03
nPCR g/kg/day	Year 0	1.26 (1.2, 1.32)	1.18 (1.11, 1.25)	1.21 (1.09, 1.33)	–	–	–	–	–	–	–	–	–
	Year –1	1.2 (1.14, 1.27)	1.18 (1.1, 1.24)	1.25 (1.13, 1.38)	–0.06 (-0.15, 0.02)	–0.002 (-0.1, 0.1)	0.04 (-0.11, 0.2)	0.06 (-0.04, 0.15)	0.24	0.005 (-0.1, 0.11)	0.93	–0.05 (-0.17, 0.06)	0.38
Albumin g/L	Year 0	37.08 (36.37, 37.8)	37.26 (36.57, 37.96)	35.42 (34.86, 35.98)	–	–	–	–	–	–	–	–	–
	Year –1	37.33 (36.54, 38.12)	37.48 (36.68, 38.27)	36.3 (35.54, 37.06)	–0.25 (-1.31, 0.82)	–0.21 (-1.27, 0.84)	–0.88 (-1.83, 0.06)	–0.13 (-1.02, 0.76)	0.77	1.59 (0.73, 2.46)	<0.001	1.72 (0.85, 2.59)	<0.001
	Year –2	36.93 (36.01, 37.85)	37.26 (36.32, 38.2)	36.97 (35.99, 37.94)	0.16 (-1, 1.32)	0.004 (-1.17, 1.17)	–1.55*^a^* (-2.68, –0.43)	–0.13 (-0.99, 0.74)	0.77	1.36 (0.52, 2.21)	0.002	1.5 (0.64, 2.35)	0.001
Albumin < 38 g/L (*n*/%)	Year 0	75 (56.8)	68 (52.7)	115 (77.7)	–	–	–	–	–	–	–	–	–
	Year –1	51 (47.2)	55 (55.6)	59 (73.8)	0.1 (-0.03, 0.22)	–0.03 (-0.16, 0.1)	0.04 (-0.08, 0.16)	–0.008 (-0.11, 0.1)	0.89	–0.24 (-0.34, –0.14)	<0.001	–0.23 (-0.34, –0.13)	<0.001
	Year –2	50 (63.3)	44 (62)	30 (61.2)	–0.07 (-0.2, 0.07)	–0.09 (-0.24, 0.05)	0.17*^a^* (0.02, 0.31)	0.001 (-0.1, 0.1)	0.98	–0.19 (-0.29, –0.09)	<0.001	–0.19 (-0.29, –0.09)	<0.001
Cr/BSA μmol/L/m^2^	Year 0	540.6 (516.7, 564.4)	543.9 (517.8, 570)	487.6 (464, 511.3)	–	–	–	–	–	–	–	–	–
	Year –1	578 (551.3, 604.8)	570.2 (540.4, 600.1)	549.5 (515.1, 583.9)	–37.45*^a^* (-73.37, –1, 53)	–26.39 (-66.06, 13.3)	–61.87*^a^* (-104, –20.15)	–3.27 (-35.85, 29.31)	0.84	52.96 (21.36, 84.56)	0.001	56.23 (24.44, 88.03)	0.001
	Year –2	556.8 (525.3, 588.3)	526.5 (489.9, 563)	601.8 (574.2, 646.4)	–16.19 (-55.74, 23.36)	17.38 (-27.55, 62.32)	–114.17*^a^* (-164.6, –63.7)	–1.84 (-34.12, 30.44)	0.91	47.94 (16.57, 79.31)	0.003	49.78 (18.19,81.36)	0.002
Cr/BSA < 380 μmol/L/m^2^ (*n*/%)	Year 0	12 (9.1)	14 (10.9)	26 (17.8)	–	–	–						
	Year –1	8 (7.6)	12 (12.1)	11 (15.9)	0.02 (-0.06, 0.09)	–0.01 (-0.1, 0.07)	0.02 (-0.09, 0.12)	–0.02 (-0.1, 0.05)	0.53	–0.09 (-0.16, –0.01)	0.03	–0.06 (-0.14, 0.01)	0.11
	Year –2	7 (9.2)	9 (13.6)	4 (9.8)	–0.001 (-0.08, 0.08)	–0.03 (-0.13, 0.07)	0.08 (-0.05, 0.21)	–0.03 (-0.1, 0.04)	0.41	–0.08 (-0.15, –0.005)	0.04	–0.05 (-0.12, 0.03)	0.21

BW, body weight; nPCR, normalized protein catabolic rate; Cr, creatinine; BSA, body surface area; *^a^p*-value < 0.05 vs. Year 0.

## Discussion

The main findings of the present study included lower serum albumin, Cr/BSA and higher rate of patients with serum albumin < 38 g/L at baseline in the group of patients with severe hyperparathyroidism (PTH ≥ 1,500 pg/ml) compared to the group with moderate hyperparathyroidism (PTH 600–1,499 pg/ml) and the group with PTH level within the target range (PTH 200–599 pg/ml). Higher PTH level was independently associated with serum albumin < 38 g/L and serum Cr/BSA < 380 μmol/L/m^2^. The analyses of longitudinal changes of nutritional parameters revealed more significant weight loss among patients with severe hyperparathyroidism. Moreover, serum albumin and Cr/BSA decreased and the proportion of patients with serum albumin < 38 g/L and serum Cr/BSA < 380 μmol/L/m^2^ increased from the preceding 1 and 2 years in the group with severe hyperparathyroidism. These changes were significant when compared to the other two groups. There was no discernable difference in the degree of nutritional impairment between patients with moderate hyperparathyroidism and patients whose PTH levels were within the target range.

The present study is the first to investigate nutritional status in patients with kidney failure with respect to the degree of hyperparathyroidism. In severe hyperparathyroidism, in addition to bone and height loss, the decrease in serum albumin and skeletal muscle mass as reflected by lower serum creatinine was evident. Comparisons of serum albumin, creatinine, body weight and height between baseline with the preceding 1 and 2 years confirmed an ongoing deterioration of nutritional status among patients with severe hyperparathyroidism. Previous small studies in patients with severe hyperparathyroidism have also described a decrease in serum albumin which improved considerably after parathyroidectomy (6–8). The cause for decreased serum albumin has yet to be fully elucidated. Inflammation in dialysis patients has multifactorial etiologies including dialysis membranes, central venous catheters, oxidative stress, immune dysfunction, bacterial products, and uremic toxins. Inflammation is a well-known cause of suppressed albumin synthesis and muscle wasting (23, 24). In the present study, NLR and PLR were high but largely similar among the three groups suggesting that nutritional impairment in severe hyperparathyroidism was not the result of heightened inflammation. Inconsistent findings regarding the relationship between hyperparathyroidism and inflammation have been reported previously. Two studies observed an association between hyperparathyroidism and increased inflammatory markers including c-reactive protein and interleukin-6, whereas the other did not find any correlations between PTH level with interleukin-1, interleukin-6 and tumor necrosis factor-alpha (8, 25, 26).

Lower serum creatinine and their progressive decline in severe hyperparathyroidism suggested the presence of ongoing muscle wasting. It is possible that, as hyperparathyroidism advances, the patients become less ambulatory leading to a reduction in the muscle mass. Sarcopenia is prevalent among patients with kidney failure. The causes include increased protein degradation and reduced protein synthesis resulting in PEW (27). A recent experimental study in 5/6 nephrectomy mice revealed the role of PTH and PTH receptor in browning of adipose tissue, increased energy expenditure, muscle wasting and cachexia. Fat-specific knockout of PTH receptor in these animals blocked adipose browning and muscle wasting (28). Another experimental study using PTH gene over-expressed mice mimicking primary hyperparathyroidism showed similar characteristics of adipose tissue browning (29). In patients with kidney failure, increased energy expenditure, reduced fat mass and muscle weakness in association with severe hyperparathyroidism have also been suggested (6, 9, 10, 30). Combining these findings with the findings from the present study, adipose tissue browning caused by excess PTH in severe hyperparathyroidism can reasonably explain the reduction in serum albumin and muscle mass. The lack of difference in the nPCR suggested that nutritional impairment was unlikely to be due to reduced dietary intake. A recent large epidemiological study observed an association between the severity of hyperparathyroidism and 12-month weight loss that appeared to be more pronounced among patients with preserved appetite (11). Another observational study did not find any changes in the nPCR after parathyroidectomy despite a significant increase in the body weight (31). In an experimental study of 5/6 nephrectomy, weight loss was associated with decreased fat and skeletal muscle mass rather than reduced food intake (28). These data suggest the inconsequential role of reduced dietary intake in nutritional impairment among patients with severe hyperparathyroidism.

Although there was no significant difference in body weight and BMI in the primary analysis, the percentage of weight reduction from the previous 2 years and the proportion of patients with ≥ 5% weight loss were more common among patients with severe hyperparathyroidism. There was also a positive correlation between BMI and serum creatinine and a negative correlation between BMI and PTH. The combined reduction in bone, muscle and fat mass was likely accountable for the decline in the body weight in severe hyperparathyroidism. The records of body weight were obtained on non-dialysis days when some patients might have started accumulating fluid. The variation in the interdialytic fluid-related weight gain of each patient inevitably interfered with the actual body weight. The reduction in serum creatinine and height was more pronounced among women. Women have less muscle and bone mass compared to men and the prevalence of premature menopause increases among women with kidney failure (32, 33). These unfavorable environments likely increased the vulnerability of musculoskeletal system of women to further insults

### Limitations of the study

Findings from a retrospective study largely reflected an association rather than a causation. To overcome some of the limitations, propensity score matching was performed to help balance the baseline characteristics and to minimize the confounders in the present study. Nutritional parameters recommended by ISRNM were adapted in the evaluation of nutritional status (15). Serum creatinine was used as a surrogate marker for muscle mass and body weight and height represented the combination of bone, muscle and fat mass. Since the body weight of hemodialysis patients in the present study was likely interfered by the fluctuation of extracellular fluid, a more accurate assessment of these compartments could be achieved by a full body composition analysis using bioelectrical impedance technology or dual energy X-ray absorptiometry in a prospective trial. Detailed data on dietary intake as well as muscle strength and performance were not available. Serum creatinine and albumin could be interfered by the presence of residual urine and proteinuria but the evaluation of residual renal function was not part of the routine patient care. However, patients in the present study had prolonged dialysis vintage with an average of 6 years and with ongoing decline in renal function, most patients were unlikely to have any significant residual renal function at the time of data collection. Patients included in the present study were relatively young with low prevalence of diabetes and cardiovascular disease. Prior to propensity score matching, patients in groups 1 and 2 were older (average age: 59 years for group 1 and 50 years for group 2) with higher prevalence of diabetes (36% in group 1 and 29% in group 2) and cardiovascular disease (27% in group 1 and 17% in group 2) compared to group 3. However, severe hyperparathyroidism in ESKD is more commonly observed among young non-diabetic patients resulting in a low frequency of elderly, diabetes and cardiovascular disease after propensity score matching (34). To study the effect of severe hyperparathyroidism among diabetics, a larger population will be required. The study population in the present study was mainly from South-Eastern Asia with small body size and low prevalence of obesity which could limit the generalizability of the findings to populations from other parts of the world.

In conclusion, patients receiving MHD with severe hyperparathyroidism had deterioration of nutritional status compared to patients with moderate hyperparathyroidism and patients with PTH level within the recommended range. The was no difference in the degree of nutritional impairment between patients with moderate hyperparathyroidism and those whose PTH levels were within the target range. These findings support the role of extreme PTH level in PEW emphasizing the importance of early and appropriate management of mineral and bone disorder.

## Data availability statement

The original contributions presented in this study are included in the article/Supplementary material, further inquiries can be directed to the corresponding author/s.

## Ethics statement

The studies involving human participants were reviewed and approved by Faculty of Medicine, Ramathibodi Hospital, Mahidol University (MURA2021/774 and MURA2017/220). Written informed consent for participation was not required for this study in accordance with the national legislation and the institutional requirements.

## Author contributions

SD conceived and designed the study, analyzed and interpreted the data, and wrote and critically revised the manuscript for important intellectual contents. All authors acquired the data, read and approved the manuscript.
