# Supplementary material for: Severe hyperparathyroidism is associated with nutritional impairment in maintenance hemodialysis patients
Source: Front Nutr. 2022 Sep 13;9:933918. doi: 10.3389/fnut.2022.933918 (PMC9513451; doi:10.3389/fnut.2022.933918)
Supplement: Supplementary file 1 [file Data_Sheet_1.PDF]

Supplementary Table 1. Pearson's correlations between nutritional and biochemical parameters

Correlations											
		**BMI**	**nPCR**	**Serum Alb**	**Serum Cr**	**Age**	**Hb**	**NLR**	**PLR**	**PTH**	**PO_4**
BMI	Pearson Correlation	1	-.099	.013	.167**	-.047	.119*	.024	-.095	-.192**	.067
	Sig. (2-tailed)		.160	.798	**.001**	.339	**.016**	.658	.078	**.000**	.174
	N	410	203	409	407	410	410	349	349	410	410
nPCR	Pearson Correlation	-.099	1	.096	.053	-.093	-.024	-.006	.056	-.036	.090
	Sig. (2-tailed)	.160		.174	.455	.187	.730	.931	.429	.613	.202
	N	203	203	202	201	203	203	202	202	203	203
Serum albumin	Pearson Correlation	.013	.096	1	.202**	-.322**	.327**	-.254**	-.137*	-.283**	.039
	Sig. (2-tailed)	.798	.174		**.000**	**.000**	**.000**	**.000**	**.011**	**.000**	.434
	N	409	202	409	406	409	409	348	348	409	409
Serum Creatinine	Pearson Correlation	.167**	.053	.202**	1	-.338**	.096	-.075	-.129*	-.224**	.401**
	Sig. (2-tailed)	**.001**	.455	**.000**		**.000**	.053	.164	**.017**	**.000**	**.000**
	N	407	201	406	407	407	407	346	346	407	407

**. Correlation is significant at the 0.01 level (2-tailed).

*. Correlation is significant at the 0.05 level (2-tailed).

Supplementary Table 2. Mixed-effects regression analyses on the differences in biochemical and nutritional parameters between baseline and the preceding 1 and 2 years

Parameters	Time	Mean (95% confidence Interval)			Within group difference from Year 0 Mean (95% confidence interval)			Between group difference Mean (95% confidence interval)					
								Comparator Group 1				Comparator Group 2	
		Group 1 200-599	Group 2 600-1499	Group 3 ≥1500	Group 1 200-599	Group 2 600-1499	Group 3 ≥1500	Group 2 600-1499	P	Group 3 ≥1500	P	Group 3 ≥1500	P
BW (kg) Male	Year 0	65.92 (62.89,68.96)	70.02 (65.6,74.44)	67.37 (64.14,70.59)	-	-	-	-	-	-	-	-	-
	Year -1	66.13 (62.99,69.27)	70.3 (65.71,74.9)	68.73 (65.07,72.39)	-0.21 (-4.57,4.16)	0.28 (-6.66,6.09)	-1.37 (-6.24,3.51)	-3.68 (-9.04,1.67)	0.18	-1.56 (-6.75,3.63)	0.55	2.12 (-3.02,7.27)	0.42
	Year -2	65.18 (61.88,68.48)	67.92 (62.75,73.09)	69.96 (65.91,74.01)	0.74 (-3.74,5.23)	2.1 (-4.7,8.9)	-2.59 (-7.77,2.58)	-4.23 (-9.45,1)	0.11	-2.72 (-7.79,2.34)	0.29	1.5 (-3.53,6.54)	0.56
BW (kg) Female	Year 0	58.31 (55.14,61.49)	55.2 (52.17,58.23)	54.43 (51.56,57.29)	-	-	-	-	-	-	-	-	-
	Year -1	58.25 (54.95,61.56)	54.87 (51.71,58.03)	55.72 (52.43,59)	0.06 (-4.52,4.645)	0.33 (-4.05,4.71)	-1.29 (-5.65,3.07)	3.12 (-1.2,7.44)	0.16	3.74 (-0.44,7.91)	0.08	0.61 (-3.66,4.89)	0.78
	Year -2	58.65 (55.18,62.12)	55.55 (52.18,58.93)	55.71 (51.94,59.47)	-0.34 (-5.04,4.37)	-0.35 (-4.88,4.19)	-1.28 (-6.01,3.46)	2.5 (-1.73,6.72)	0.25	3.28 (-0.82,7.38)	0.12	0.79 (-3.41,4.99)	0.71
Height (cm) Male	Year 0	165.4 (163.3,167.5)	165.9 (163.9,168)	165.7 (163.8,167.6)	-	-	-	-	-	-	-	-	-
	Year -1	165.9 (163.5,168.2)	165.6 (163.5,167.8)	166.3 (163.9,168.7)	-0.47 (-3.61,2.67)	0.3 (-2.65,3.26)	-0.55 (-3.59,2.5)	-0.24 (-2.6,2.11)	0.84	1 (-2.18,2.37)	0.93	0.34 (-1.92,2.6)	0.77
	Year -2	165.5 (163,168)	166.9 (164.4,169.3)	168 (165.4,170.6)	-0.12 (-3.39,3.15)	-0.93 (-4.13,2.26)	-2.28 (-5.51,0.95)	-0.68 (-3.25,1.89)	0.6	-0.29 (-2.81,2.23)	0.82	0.39 (-2.1,2.88)	0.76
Height (cm) Female	Year 0	156.7 (154.5,158.9)	156.8 (154.4,159.2)	153.7 (152,155.4)	-	-	-	-	-	-	-	-	-
	Year -1	157.1 (154.8,159.4)	155.3 (152.6,157.9)	154.3 (152.4,156.1)	-0.37 (-3.57,2.83)	1.53 (-2.05,5.11)	-0.55 (-3.03,1.92)	2.28 (-0.25,4.8)	0.08	4.55 (2.12,6.99)	<0.001	2.28 (-0.22,4.77)	0.07
	Year -2	158.3 (155.6,160.9)	156.3 (153.4,159.1)	156.1 (153.9,158.3)	-1.57 (-5.02,1.88)	0.56 (-3.13,4.24)	-2.4 (-5.13,0.33)	0.94 (-1.82,3.69)	0.5	2.91 (0.28,5.55)	0.03	1.98 (-0.76,4.71)	0.16
BMI (kg/m^2) Male	Year 0	23.72 (22.76,24.68)	24.97 (23.57,26.37)	24.37 (23.3,25.43)	-	-	-	-	-	-	-	-	-
	Year -1	23.92 (22.93,24.92)	25.06 (23.61,26.51)	24.78 (23.58,25.99)	-0.2 (-1.58,1.18)	-0.09 (-2.11,1.92)	-0.42 (-2.03,1.2)	-1.21 (-2.91,0.49)	0.16	-0.57 (-2.22,1.08)	0.5	0.64 (-0.99,2.28)	0.44
	Year -2	23.58 (22.53,24.63)	24.33 (22.7,25.96)	24.93 (23.59,26.27)	0.14 (-1.28,1.56)	0.64 (-1.51,2.79)	-0.56 (-2.28,1.15)	-1.33 (-2.98,0.32)	0.11	-0.81 (-2.41,0.79)	0.32	0.52 (-1.07,2.11)	0.52
BMI (kg/m^2) Female	Year 0	23.18 (22.11,24.26)	22.5 (21.43,23.58)	23.01 (21.89,24.14)	-	-	-	-	-	-	-	-	-
	Year -1	23.2 (22.08,24.32)	22.31 (21.19,23.43)	23.32 (22.03,24.61)	-0.02 (-1.57,1.54)	0.19 (-1.36,1.74)	0.31 (-2.02,1.41)	0.66 (-0.9,2.21)	0.41	0.1 (-1.4,1.61)	0.89	-0.55 (-2.09,0.99)	0.48
	Year -2	23.36 (22.18,24.54)	22.53 (21.33,23.73)	22.92 (21.43,24.4)	-0.18 (-1.77,1.42)	-0.03 (-1.64,1.58)	0.1 (-1.76,1.96)	0.59 (-0.95,2.13)	0.45	0.17 (-1.32,1.66)	0.82	-0.42 (-1.95,1.11)	0.59
	Year 0	6 (9.2)	6 (9)	7 (9.3)	-	-	-	-	-	-	-	-	-

Cr/BSA <380 µmol/L/m^2 Male	Year -1	4 (7.8)	5 (8.9)	2 (5.9)	0.01 (-0.09,0.12)	0.00 (-0.1,0.1)	0.04 (-0.08,0.15)	0.002 (-0.09,0.1)	0.97	0.01 (-0.08,0.11)	0.81	0.01 (-0.08,0.1)	0.83
	Year -2	3 (8.6)	1 (3.2)	1 (4.8)	0.007 (-0.11,0.12)	0.06 (-0.06,0.17)	0.05 (-0.09,0.18)	-0.12 (-0.08,0.11)	0.8	0.007 (-0.08,0.1)	0.87	-0.005 (-0.1,0.09)	0.92
Cr/BSA <380 µmol/L/m^2 Female	Year 0	6 (9)	8 (12.9)	19 (26.8)	-	-	-	-	-	-	-	-	-
	Year -1	7 (7.4)	7 (16.3)	9 (25.7)	0.02 (-0.09,0.12)	-0.03 (-0.18,0.11)	0.01 (-0.17,0.19)	-0.05 (-0.17,0.06)	0.37	-0.18 (-0.3,-0.07)	0.002	-0.13 (-0.25,-0.02)	0.03
	Year -2	4 (9.8)	8 (22.9)	3 (15)	-0.008 (-0.12,0.1)	-0.1 (-0.26,0.06)	0.12 (-0.1,0.34)	-0.07 (-0.18,0.04)	0.2	-0.17 (-0.27,-0.06)	0.003	-0.1 (-0.21,0.02)	0.09
